# Preliminary Impact of a Social Connection Program on Older Adults

**DOI:** 10.1093/geroni/igab046.3317

**Published:** 2021-12-17

**Authors:** Rachel Ungar, Rifky Tkatch, Yan Cheng, Sandra Kraemer, Michael McGinn, Ellen Wicker

**Affiliations:** 1 UnitedHealth Group, Minnetonka, Minnesota, United States; 2 UnitedHealth Group, Oak Park, Michigan, United States; 3 UnitedHealthcare, Minnetonka, Minnesota, United States; 4 AARP Services, Inc., Washington, District of Columbia, United States

## Abstract

Background: Research demonstrates social connections decrease loneliness and improves life satisfaction among older adults. Unfortunately, the COVID-19 pandemic has limited social connectedness, specifically for older adults. Thus, programs aiming to increase social connectedness among older adults are imperative. Purpose: The primary objective of this study was to determine if the telephonic Peer-to-Peer (P2P) program can improve social connectedness and loneliness among older adults. A secondary objective was to determine whether additional improvements in life satisfaction and perception of aging were achieved. Methods: Eligible older adults (age 65+) were recruited via outbound calls and/or a mailer. Participants were mailed a T1 survey, completed intervention training, and matched into a dyad. The matched dyad engaged in weekly telephone calls for 12 weeks. Post 12 weeks, participants completed a T2 survey, and a T3 four weeks later. Results: Overall, 7,544 individuals were contacted to participate, and 759 expressed interest in participation. A total of 475 participants (62%) completed a T1, 372 (78%) completed training, and 348 (94%) were matched. Gender distribution was skewed towards females (74%), and most were 65-74 years old (53%). Preliminary results show significant differences between lonely and not lonely participants, with lonely participants reporting more negative health associations across all measures. Conclusion: Once agreeing to participate, results showed a high likelihood of continuing in P2P, thus demonstrating a social connectedness opportunity for older adults. Delay in mailing and scheduling training may contribute to challenges in attrition. However, developing automated processes utilizing technology may decrease lag time for future phases.

